# The impact of lung parenchyma attenuation on nodule volumetry in lung cancer screening

**DOI:** 10.1186/s13244-021-01027-0

**Published:** 2021-06-25

**Authors:** Diana Penha, Erique Pinto, Bruno Hochhegger, Colin Monaghan, Edson Marchiori, Luís Taborda-Barata, Klaus Irion

**Affiliations:** 1 Universidade da Beira Interior Faculdade de Ciências da Saúde, Covilha, Portugal; 2 Liverpool Heart and Chest Hospital NHS Foundation Trust, Liverpool, UK; 3 Pontificia Universidade Catolica do Rio Grande do Sul, Porto Alegre, Brazil; 4 Universidade Federal do Rio de Janeiro Faculdade de Medicina, Rio de Janeiro, RJ Brazil; 5 Universidade Federal Fluminense Faculdade de Medicina, Niterói, RJ Brazil; 6 Manchester University NHS Foundation Trust, Manchester, UK

**Keywords:** Lung cancer screening, Volumetry, Segmentation, Interstitial lung disease

## Abstract

**Background:**

Recent recommendations for lung nodule management include volumetric analysis using tools that present intrinsic measurement variability, with possible impacts on clinical decisions and patient safety. This study was conducted to evaluate whether changes in the attenuation of the lung parenchyma adjacent to a nodule affect the performance of nodule segmentation using computed tomography (CT) studies and volumetric tools.

**Methods:**

Two radiologists retrospectively applied two commercially available volumetric tools for the assessment of lung nodules with diameters of 5–8 mm detected by low-dose chest CT during a lung cancer screening program. The radiologists recorded the success and adequacy of nodule segmentation, nodule volume, manually and automatically (or semi-automatically) obtained long- and short-axis measurements, mean attenuation of adjacent lung parenchyma, and presence of interstitial lung abnormalities or disease, emphysema, pleural plaques, and linear atelectasis. Regression analysis was performed to identify predictors of good nodule segmentation using the volumetric tools. Interobserver and intersoftware agreement on good nodule segmentation was assessed using the intraclass correlation coefficient.

**Results:**

In total, data on 1265 nodules (mean patient age, 68.3 ± 5.1 years; 70.2% male) were included in the study. In the regression model, attenuation of the adjacent lung parenchyma was highly significant (odds ratio 0.987, *p* < 0.001), with a large effect size. Interobserver and intersoftware agreement on good segmentation was good, although one software package performed better and measurements differed consistently between software packages.

**Conclusion:**

For lung nodules with diameters of 5–8 mm, the likelihood of good segmentation declines with increasing attenuation of the adjacent parenchyma.

## Key points

- Lung nodule volumetry is currently recommended for lung nodule management.
- Artificial intelligence tools for volumetric analysis still present with some limiting factors.
- Location, size, shape, density are the most common factors affecting nodule volumetry.
- Attenuation of the lung parenchyma is another limiting factor for nodule volumetry.
- Recognition of these factors has impact on clinical decisions and patient safety.

## Background

Although lung nodule management guidelines historically have recommended the measurement of nodules using electronic calipers, artificial intelligence tools are increasingly used for nodule detection and measurement. This shift was introduced mainly in the Dutch–Belgian lung cancer screening (*Nederlands–Leuvens Longkanker Screenings Onderzoek* or NELSON) trial, and was subsequently integrated into the guidelines of the Fleischner Society and British Thoracic Society for incidental pulmonary nodules with volumes exceeding 100 and 80 mm^3^, respectively. These guidelines clearly identify micronodules (< 5 mm) as benign, and larger nodules (> 8 mm) as having a high risk of malignancy, as supported by data from the NELSON trial. For nodules between 5 and 8 mm, growth rate is a better discriminator between benign and malignant lesions than size or morphological characteristics [1, 2].

The recommendation that pulmonary nodules be measured volumetrically is based on the recognition that nodule diameter does not accurately reflect size or growth, as not all nodules are perfectly spherical or symmetrically growing. Thus, the calculation of nodule volume enables the use of better growth markers, such as the volume doubling time (VDT) [3].

Several recent studies have examined the reliability and limiting factors of pulmonary nodule volumetry, such as location (i.e., adjacency or connection to high-density structures), size, shape, and density [4–6]. Marked volumetric variability among studies for nodules smaller than 6 mm in diameter, and the high probability that the segmentation of ground-glass nodules with currently available software will fail, have been recognized [7, 8]. Technical factors, such as the number of detectors in the computed tomography (CT) scanner, administration of contrast medium, slice thickness, interpolation of reconstructed images, and reconstruction algorithm used, also affect the accuracy of volumetry [9–12].

However, little is known about the impact of changes in the density of adjacent lung parenchyma on the volumetric evaluation of a lung nodule; such changes may alter the degree of contrast between these structures. Empirically, an increase in contrast caused by certain pathological conditions (e.g., emphysema) is assumed to reduce the variability of volume measurement, whereas a decrease in contrast [e.g., due to interstitial lung disease (ILD)] is thought to increase this variability [12]. Data from lung cancer screening programs suggest that the prevalence of ILD is as high as 20% [13]. Effects on nodular volume calculation attributable to changes in the attenuation of the adjacent pulmonary parenchyma would have medical and therapeutic implications for a substantial number of patients and major financial impacts on lung cancer screening programs. This study was conducted to evaluate the effect of the degree of contrast of the parenchyma adjacent to a pulmonary nodule on nodule segmentation using volumetric software.

## Materials and methods

The Institutional Research Committee Review Board approved this retrospective cross-sectional study (observational, analytical) and waived the requirement for written informed consent due to the use of existing clinical data.

### Study sample

The study sample was derived from all patients participating in a lung cancer screening program in a tertiary hospital in Northeastern England between August 2016 and December 2018. All CT screening examinations were performed with the same equipment (Somatom Definition Flash; Siemens, Erlangen, Germany) using a low-dose CT protocol (Table 1). All CT studies without technical (e.g., respiratory motion) artifacts showing solid pulmonary nodules with diameters of 5–8 mm were included in this study. For the included patients, the clinical records were accessed via the hospital information system, and the following patient data were collected: patient age and sex and previous histories of chronic obstructive pulmonary disease (COPD), tuberculosis (TB), and lung surgery (Table 2).

**Table 1:** Low-dose chest CT imaging protocol parameters

Range	Lung apices–bases
Respiratory phase	Inspiration, breath hold
Enhancement	None
Image reconstruction	2-mm thickness, 1-mm overlap
Kernels	B60f sharp/lung, B30f medium smooth/lung, B20f smooth/mediastinum
Acquisition parameters	kVp and mAs varied according to body habitus
Planned CTDI(vol)	2.03 mGy, with 120 kVp and quality reference of 30 mAs

CT, computed tomography

**Table 2:** Patients’ demographic and clinical characteristics

Characteristic	*n* (%) or mean ± SD (*n* = 1265)
Age (years)	68.3 ± 5.1
Sex	
Male	888 (70.2)
Female	377 (29.8)
Previous lung surgery	
No	1238 (97.9)
Yes	27 (2.1)
Chronic obstructive pulmonary disease	
No	1198 (94.7)
Yes	67 (5.3)
Tuberculosis	
No	1258 (99.4)
Yes	7 (0.6)

SD, standard deviation

### Readers and measurements

**Fig. 1:**
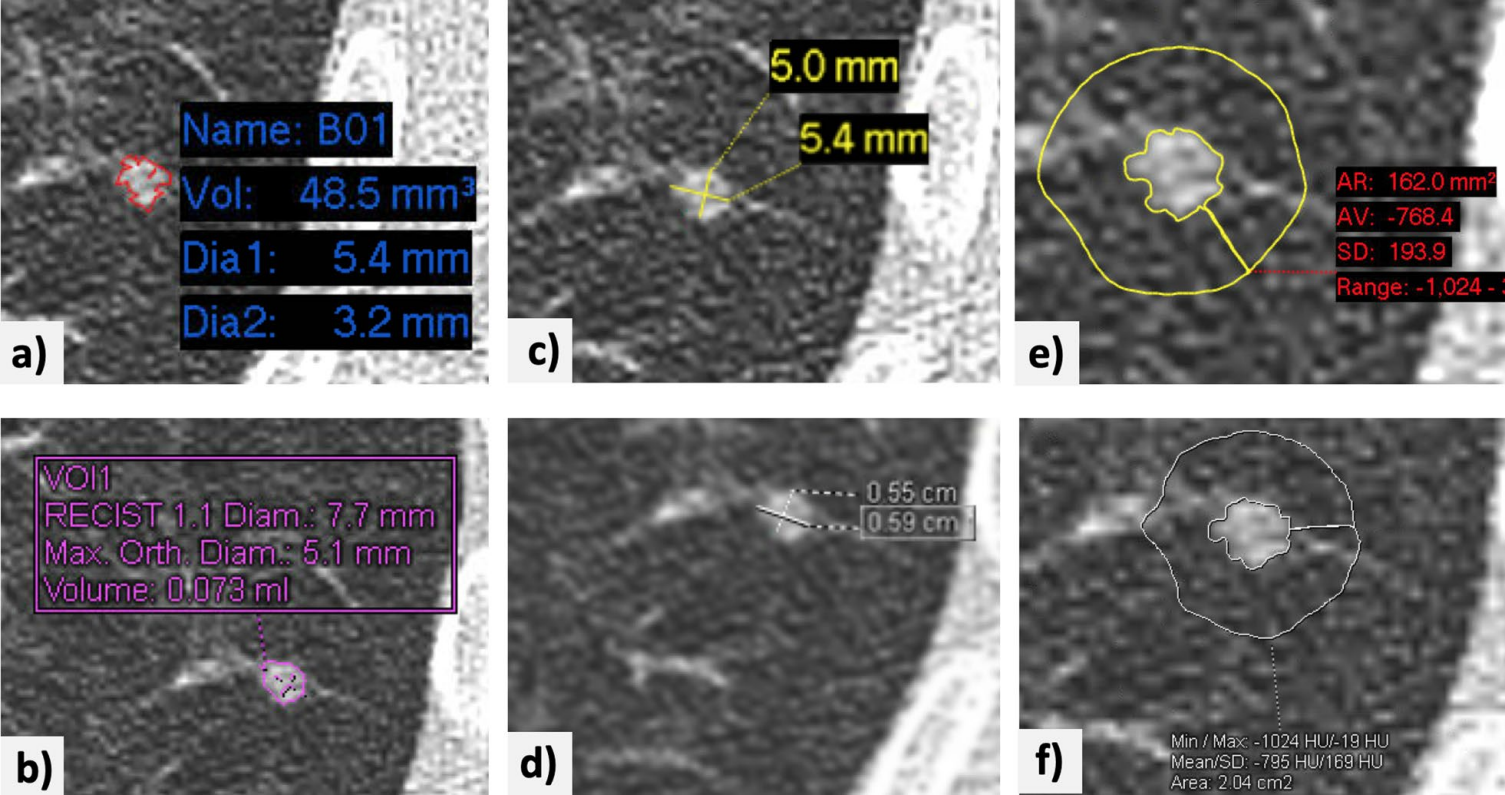
Example of the implementation of the nodule measurement protocol. A small nodule is identified in the posterior segment of the left lower lobe of the lung. **a**, **b** The volumetric tools [Vue PACS, ver. 11.4.01.1011; Carestream^©^ (tool 1) and Syngo via VB20, Siemens^©^ (tool 2)] are used to segment the nodule, yielding volumes of 48.5 and 75 mm^3^, respectively. **c**, **d** The longest orthogonal diameters are measured manually using electronic calipers tools in both software packages. **e**, **f** A region of interest (5-mm thickness) is drawn manually around the nodule for determination of the average attenuation of adjacent lung parenchyma (− 768.4 and − 795 HU obtained with tools 1 and 2, respectively). The images have been edited to improve the readability of the measurements

Two cardiothoracic radiologists with 5 (reader 1) and 10 (reader 2) years of experience, respectively, identified and measured the pulmonary nodules, following the protocol described in Fig. 1 and using the Carestream Vue PACS v 11.4.01.1011 (Carestream Health, Inc, Rochester, NY; tool 1) and Syngo via VB20 (Siemens Healthineers AG, Erlangen, Germany; tool 2) volumetric software packages. Disagreements among readers regarding the inclusion of a pulmonary nodule were resolved by consensus after a discussion between both readers and a third chest radiologist with more than 25 years of experience (consensus decision).

**Fig. 2:**
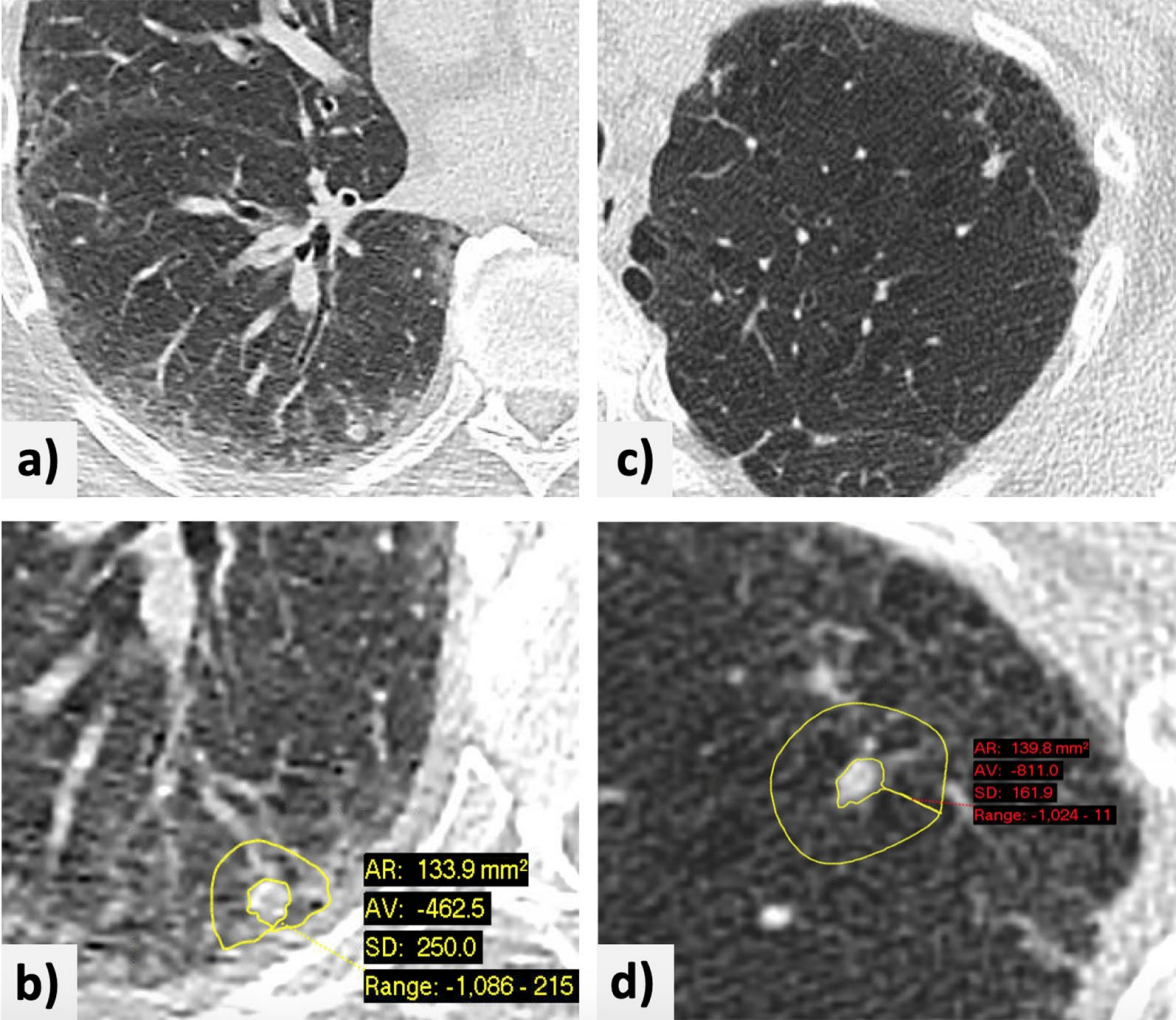
Examples of the measurement of lung nodules in lung parenchyma with attenuation changes. **a** A small subpleural nodule in the right lower lobe of the lung of a patient with known interstitial lung disease. **b** The nodule is shown with a region of interest drawn manually around it, with a rim of about 5-mm thickness. The average attenuation of the adjacent lung parenchyma on this slice is − 462.5 HU. **c** A nodule in the anterolateral aspect of the left upper lobe of the lung in a patient with known centrilobular and paraseptal emphysema. **d** Manual measurement of the average attenuation of the surrounding lung parenchyma (− 811 HU)

For each nodule identified, the readers used both software packages to record the following:

- Nodule segmentation success or failure (whether the software tool provided a result or notified the user of measurement failure). Failure was defined as three consecutive failed attempts at segmentation.
- Nodule segmentation adequacy or inadequacy (in case of segmentation success, this is subjective impression by the reader of full nodule inclusion and with vessel and parenchymal consolidation exclusion).
- Nodule volume, calculated semi-automatically with the software.
- Long- and short-axis nodule diameters (orthogonal and in the axial plane), determined manually with electronic calipers, rounded to one decimal place.
- *‘Mean attenuation of the adjacent lung parenchyma’*, in Hounsfield units, obtained after using the PACS region-of-interest (ROI) tool to delineate an area of about 5 mm thickness surrounding the nodule, rounded to one decimal place (Fig. 2)
- Presence or absence of signs suggestive of interstitial lung abnormalities (ILA) or ILD, emphysema, pleural plaques, and linear atelectasis.

### Statistical analysis

The clinical and imaging data were analyzed using SPSS software (ver. 26.0; IBM Corporation, Armonk, NY, USA). The dichotomous variable *‘Proper segmentation’*, reflecting segmentation success and adequacy, and the continuous variable *‘Average of long and short diameters’*, reflecting the average of the nodule’s long- and short-axis, manually measured, diameters (following the Fleischner Society recommendation [1]), were created and values were calculated for all included cases.

A descriptive statistical analysis is performed including sample mean, standard deviation (SD), minimum, maximum and quartiles (Table 3).

**Table 3:** Results for quantitative variables

Variable	Reader	Tool	*n*	Mean ± SD	Min	Q1	Q2	Q3	Max
Volume (cm^3^)	Global		5030	98.9 ± 193.2	0.0	43.5	67.0	100.0	8200.0
	Reader 1	Tool 1	1250	102.7 ± 257.7	3.0	43.9	67.4	100.0	8200.0
		Tool 2	1264	97.1 ± 105.5	0.0	42.0	66.5	110.0	1401.0
	Reader 2	Tool 1	1251	100.3 ± 250.6	1.3	44.0	67.2	100.0	8200.0
		Tool 2	1265	95.5 ± 98.5	0.0	43.0	66.0	110.5	1402.0
Manual long-axis diameter (mm)	Global		5060	6.19 ± 1.12	4.0	5.3	6.1	7.0	10.0
	Reader 1	Tool 1	1265	6.13 ± 1.11	4.4	5.2	6.0	7.0	8.4
		Tool 2	1265	6.27 ± 1.11	4.2	5.4	6.2	7.1	9.3
	Reader 2	Tool 1	1265	6.11 ± 1.12	4.0	5.2	6.0	7.0	9.0
		Tool 2	1265	6.26 ± 1.11	4.0	5.3	6.1	7.1	10.0
Manual short-axis diameter (mm)	Global		5060	4.68 ± 1.00	1.9	4.0	4.6	5.3	9.1
	Reader 1	Tool 1	1265	4.62 ± 1.00	2.2	3.9	4.5	5.2	8.3
		Tool 2	1265	4.74 ± 1.00	2.3	4.1	4.6	5.3	9.1
	Reader 2	Tool 1	1265	4.63 ± 1.00	1.9	3.9	4.5	5.2	8.5
		Tool 2	1265	4.75 ± 0.99	2.6	4.1	4.6	5.3	8.4
‘Average of long and short diameters’ (mm)	Global		5060	5.44 ± 0.95	3.2	4.7	5.3	6.1	9.2
	Reader 1	Tool 1	1265	5.38 ± 0.96	3.4	4.7	5.2	6.0	8.3
		Tool 2	1265	5.51 ± 0.94	3.6	4.8	5.4	6.2	9.2
	Reader 2	Tool 1	1265	5.37 ± 0.96	3.2	4.7	5.2	6.0	8.8
		Tool 2	1265	5.51 ± 0.95	3.6	4.8	5.4	6.2	8.7
*‘Mean attenuation of the adjacent lung parenchyma’* (HU)	Global		5060	− 774.2 ± 84.7	− 937.0	− 833.0	− 790.2	− 735.8	− 296.0
	Reader 1	Tool 1	1265	− 761.1 ± 85.3	− 933.4	− 821.0	− 775.2	− 722.5	− 306.8
		Tool 2	1265	− 787.8 ± 82.4	− 932.0	− 845.0	− 804.0	− 752.0	− 317.0
	Reader 2	Tool 1	1265	− 760.3 ± 84.7	− 925.9	− 819.9	− 775.4	− 722.4	− 315.1
		Tool 2	1265	− 787.5 ± 82.0	− 937.0	− 845.0	− 805.0	− 749.5	− 296.0

SD, standard deviation; Min, minimum; Q, quartile; max, maximum

The data were analyzed using a binary logistic regression model, with *‘Proper segmentation’* serving as the dependent variable and *‘Average of long and short diameters’*, *‘Mean attenuation of the adjacent lung parenchyma’*, reader, software package, patient age and sex, and relevant epidemiological factors (previous lung surgery, ILAs/ILD, emphysema, COPD, TB, calcified pleural plaques, and linear atelectasis; reference = absent for all variables) serving as independent variables (predictors). Automatic selection of the significant independent variables was performed (significance threshold of 0.10). The Nagelkerke *R*^2^ value was used to assess how much of the variance of dependent variable (*‘Proper segmentation’*) is explained by the independent variables. The Hosmer–Lemeshow chi-squared goodness-of-fit test and the omnibus test of model coefficients were used to assess the overall fit of the model. Analysis of variance between readers and software packages was performed using the one-way ANOVA test. The intraclass correlation coefficient (ICC) and an absolute agreement–type two-way mixed model were used to assess interobserver and intersoftware agreement.

## Results

**Fig. 3:**
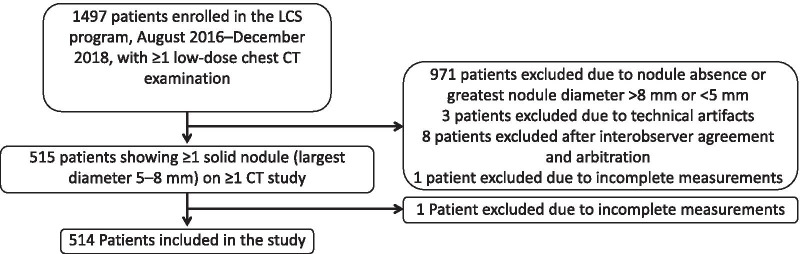
Flow chart of patient selection and inclusion. LCS, lung cancer screening; CT, computed tomography

One thousand four hundred and ninety-seven participants were identified as being enrolled in the screening program between August 2016 and December 2018, and having at least one low-dose chest CT examination during this period of time. Some participants had additional low-dose CT scans performed under this LCS program outside of this time frame, and these were also included in the study. The earliest scan dated from 5th April 2016 and the latest from 2nd August 2020. Data from 971 patients were excluded due to the absence of qualifying lung nodules, data from three patients were excluded due to respiratory motion artifacts, and data from eight patients were excluded after consensus decision. One additional patient was excluded due to technical issue specific to one software package that failed to access the patient’s records. The final sample consisted of 5060 measurements (1265/observer/software package) taken on CT studies of 514 patients (Fig. 3). The patients’ demographic and clinical characteristics are summarized in Table 2.

Readers 1 and 2 recorded *‘Proper segmentation’* (defined as success and adequacy of the segmentation) more frequently with tool 2 (88.1% and 88.4%, for reader 1 and reader 2 respectively) than with tool 1 (84.8% and 83.8%, for reader 1 and reader 2, respectively).

For readers 1 and 2, the mean nodule volumes (cm^3^) obtained using tool 1 (102.7 ± 257.7 and 100.3 ± 250.6, for reader 1 and reader 2 respectively) were greater than those obtained using tool 2 (97.1 ± 105.5 and 95.5 ± 98.5, for reader 1 and reader 2 respectively). For both tools, the volumes recorded by reader 1 were greater than those recorded by reader 2. Both readers also recorded greater *‘Average of long and short diameters’* (mm) values with tool 2 (5.51 ± 0.94 and 5.51 ± 0.95, for reader 1 and reader 2 respectively) than with tool 1 (5.38 ± 0.96 and 5.37 ± 0.96, for reader 1 and reader 2 respectively). *‘Average of long and short diameters’* values obtained with each software package were similar between readers. Both readers obtained greater *‘Mean attenuation of the adjacent lung parenchyma’* values (Hounsfield Units; HU) with tool 1 (− 761.1 ± 85.3 and − 760.3 ± 84.7, for reader 1 and reader 2 respectively) than with tool 2 (− 787.8 ± 82.4 and − 787.5 ± 82.0, for reader 1 and reader 2 respectively). *‘Mean attenuation of the adjacent lung parenchyma’* values obtained with each software package were similar between readers (Table 3).

The binary logistic regression model included data from 5030 valid cases, after the exclusion of 30 cases with missing values. The Hosmer–Lemeshow test verified the goodness of model fit (*χ*^2^_8_ = 15.23, *p* = 0.055) and the omnibus test indicated that the model with predictors differed significantly from the model with only the intercept (*χ*^2^_5_ = 1601.47, *p* < 0.001). The Nagelkerke *R*^2^ value indicated that the model explained 50.3% of the variation in the dependent variable.

The odds of *‘Proper segmentation’* increased by a factor of 1.558 (95% confidence interval (CI), 1.350–1.797) with each 1-mm increase in *‘Average of long and short diameters’* (*p* < 0.001) and by a factor of 3.414 (95% CI 1.575–7.401) with a previous history of lung surgery (*p* = 0.002); they decreased by a factor of 0.984 (95% CI 0.982–0.986) with each 1-mm^3^ increase in nodule volume (*p* < 0.001), by a factor of 0.987 (95% CI 0.985–0.988) with each Hounsfield-unit (HU) increase in *‘Mean attenuation of the adjacent lung parenchyma’* (*p* < 0.001), and by a factor of 0.593 (95% CI 0.414–0.849) in the presence of calcified pleural plaques (*p* = 0.004). No other variable significantly predicted *‘Proper segmentation’* (Table 4). The effect size was greatest for *‘Mean attenuation of the adjacent lung parenchyma’* (*ζ*^2^ = 0.195), followed by nodular volume (*ζ*^2^ = 0.033).

**Table 4:** Parameter estimates for the prediction of nodule segmentation success and adequacy

Variable	OR	95% CI	*p*	Effect size
‘Average of long and short diameters’	1.558	1.350–1.797	***< 0.001	0.006
Volume	0.984	0.982–0.986	***< 0.001	0.033
*‘Mean attenuation of the adjacent lung parenchyma’*	0.987	0.985–0.988	***< 0.001	0.195
Previous lung surgery	3.414	1.575–7.401	**0.002	0.000
Pleural plaques	0.593	0.414–0.849	**0.004	0.002

Excluded variables	*p*
Observer	0.584
Software	0.385
Age	0.083
Sex	0.875
ILA/ILD	0.488
Emphysema	0.169
COPD	0.952
Tuberculosis	0.401
Linear atelectasis	0.096

OR, odds ratio; CI, confidence interval; ***p < 0.001; **p < 0.01

ILA, interstitial lung abnormality; ILD, interstitial lung disease; COPD, chronic obstructive pulmonary disease

ICCs for the whole sample and tools 1 and 2 (0.905 (95% CI 0.897–0.912), 0.885 (95% CI 0.872–0.897), and 0.929 (95% CI 0.920–0.936), respectively) indicated very high intersoftware reliability, and greater reliability of tool 2 than of tool 1. Analysis of variance (ANOVA) revealed no significant difference between readers for the whole sample (*F*_1,2519_ = 0.962, *p* = 0.327), tool 1 (*F*_1,1264_ = 2.452, *p* = 0.118), or tool 2 (*F*_1,1264_ = 0.257, *p* = 0.621). Similarly, ICCs (0.745 (95% CI 0.722–0.766), 0.741 (95% CI 0.710–0.769), and 0.749 (95% CI 0.717–0.778), for the whole sample, reader 1 and reader 2, respectively) indicated reasonable interobserver reliability, with no significant difference between readers. ANOVA revealed significant differences between software packages for the whole sample (*F*_1,2519_ = 41.642, *p* < 0.001), reader 1 (*F*_1,1264_ = 14.615, *p* < 0.001), and reader 2 (*F*_1,1264_ = 28.166, *p* < 0.001).

## Discussion

This study showed that the probability of proper segmentation of lung nodules with diameters of 5–8 mm is related mainly to the *‘Mean attenuation of the adjacent lung parenchyma’*, followed by nodule volume and the *‘Average of long and short diameters’*. Given the global variability of *‘Mean attenuation of the adjacent lung parenchyma’*, this finding could have substantial clinical implications.

The results of this study indicate that the probability of proper segmentation using volumetric software is reduced for smaller nodules. This finding is in line with previous reports that smaller nodules exhibit greater volumetric variability (up to 30% for nodules with diameters < 6 mm) [14, 15]. In this context, the decreased probability of proper segmentation with increasing nodule volume (which is related to nodule diameter) is counterintuitive. This finding may be explained by the fact that the automatic calculation of nodule volume is reliant on the volumetric tool’s algorithm, while the variable *‘Average of long and short diameters’* is calculated from the manually measured long- and short-axis diameters of the nodule, and as such, describes the observer’s assessment of the nodule. Since an inadequate nodule segmentation is likely to involve over segmentation and overestimation of nodule volume, this inverse correlation between volume with proper segmentation may reflect an increase in the error of measurement by the volumetric tool.

A previous history of lung surgery and the presence of calcified pleural plaques were also related significantly to proper segmentation in this study, although their effect sizes were negligible. Previous lung surgery increased the probability of proper segmentation, possibly because partial and total pneumonectomies promote major changes in vascular and respiratory mechanisms via compensatory overexpansion of the remaining lung, and possibly via hormonally regulated compensatory growth of the remaining lung lobes in the attempt to restore normal mass, structure, and function [16–18]. To our knowledge, however, the literature contains no report on changes lung parenchyma attenuation after lung surgery, and the negligible effect size and small number of patients with previous histories of lung surgery in our sample should caution against over interpretation. The presence of pleural plaques reduced the probability of proper segmentation, possibly due to the architectural distortion of the lung parenchyma that it causes.

The dataset used in this study did not contain information about the proximity of the measured nodules to focal parenchymal changes, such as surgical scars or pleural plaques, which renders interpretation difficult and the model incomplete. This factor could also help to explain the nonsignificant effect of ILA/ILD-related changes in our model, despite the clear effect of the average attenuation of the lung parenchyma adjacent to the nodule and the increased lung parenchymal attenuation caused by ILA/ILD [19–22].

A cutoff of -950 HU is the most widely accepted threshold in quantitative analysis for distinguishing emphysema from normal lung tissue [23–25]. This threshold is based on the routine full-dose chest CT protocol used in clinical practice. All values in our sample exceeded this threshold, regardless of the presence of emphysema, which could be related to the lower signal-to-noise ratio of the low-dose protocol used in screening; and/or the nodule itself may distort the parenchyma and influence its surrounding attenuation. As far as the authors are aware, no specific threshold has been defined for low-dose protocols. We suspect that it would differ from that used for full-dose protocols, but more evidence is needed.

Our analysis of mean values revealed that for automatic measurements there were differences in volume measurements between software packages and readers, but for manual measurements (long and short-diameter measurements and attenuation of the lung parenchyma adjacent to the nodule) there were only differences between software packages (not between readers). We also found good performance in terms of interobserver and intersoftware reliability, although less so for the latter, in line with the current recommendation that follow-up studies performed in the context of lung cancer screening programs be reported by the same reader and performed using the same software package as the baseline study. These findings also suggest that the manual measurements of short- and long-axis diameters are more reliable among readers than the volumetric tools’ automatic measurements.

The present study was conducted with a large sample of nodule measurements; larger, to our knowledge, than any other published series. However, it has several limitations; notably, the use of a nonstandard measurement of lung parenchymal attenuation (selected as a reasonable compromise, as no standard exists) and lack of information on the location of focal parenchymal changes (i.e., pleural plaques and changes resulting from previous lung surgery) relative to lung nodule location. Future research could further examine the effects of nodule size on the results found (is the impact of the average attenuation of the lung parenchyma adjacent to the nodule in the nodule segmentation more significant in smaller nodules?), and how it effects the calculation of a nodule’s VDT (is the VDT a reliable indicator of nodule’s growth in nodules with abnormal average attenuation of the lung parenchyma adjacent to the nodule?).

## Conclusion

For lung nodules measuring between 5 and 8 mm in long-axis diameter, an increase in the average attenuation of the adjacent lung parenchyma is related to a decrease in the quality of the nodule’s segmentation by volumetric tools, contributing to measurement error. When following lung nodules in the setting of abnormal lung parenchymal attenuation, care should be taken when interpreting automatic measurements of the nodule to assess growth.

## Data Availability

The datasets used and/or analyzed during the current study are available from the corresponding author on reasonable request.

## Abbreviations

ANOVA: Analysis of variance
CI: Confidence interval
COPD: Chronic obstructive pulmonary disease
CT: Computed tomography
HU: Hounsfield unit
ICC: Interclass correlation coefficient
ILA: Interstitial lung abnormality
ILD: Interstitial lung disease
TB: Previous tuberculosis
VDT: Volume doubling time
